# Connecting Hospitalized Patients with Their Families: Case Series and Commentary

**DOI:** 10.1155/2011/804254

**Published:** 2011-10-23

**Authors:** Kourosh Parsapour, Alexander A. Kon, Madan Dharmar, Amy K. McCarthy, Hsuan-Hui Yang, Anthony C. Smith, Janice Carpenter, Candace K. Sadorra, Aron D. Farbstein, Nayla M. Hojman, Gary L. Wold, James P. Marcin

**Affiliations:** ^1^Department of Pediatrics, Miller Children's Hospital, Long Beach, CA 90806, USA; ^2^Department of Pediatrics, Naval Medical Center San Diego, San Diego, CA 92134, USA; ^3^Department of Pediatrics, University of California Davis, Sacramento, CA 95817, USA; ^4^Centre for Online Health, The University of Queensland, Herston, QLD 4029, Australia; ^5^Center for Health and Technology, University of California Davis, Sacramento, CA 95817, USA; ^6^Pediatric Critical Care Medicine, UC Davis Children's Hospital, 2516 Stockton Boulevard, Sacramento, CA 95817, USA

## Abstract

The overall aim of this project was to ascertain the utilization of a custom-designed telemedicine service for patients to maintain close contact (via videoconference) with family and friends during hospitalization. We conducted a retrospective chart review of hospitalized patients (primarily children) with extended hospital length of stays. Telecommunication equipment was used to provide videoconference links from the patient's bedside to friends and family in the community. Thirty-six cases were managed during a five-year period (2006 to 2010). The most common reasons for using Family-Link were related to the logistical challenges of traveling to and from the hospital—principally due to distance, time, family commitments, and/or personal cost. We conclude that videoconferencing provides a solution to some barriers that may limit family presence and participation in care for hospitalized patients, and as a patient-centered innovation is likely to enhance patient and family satisfaction.

## 1. Introduction

Admission to a hospital ward or Intensive Care Unit (ICU) is a significant stressor for both the patient and their family [1]. Not only do the patient and family have many fears and concerns that surround the illness, surgery, or trauma instigating the admission, but also they are forced to cope with unfamiliar environments in which they have little control. The hospitalization affects not only the patient, but also the network of family, friends, and community.

Additional stressors include obstacles and limitations to visitation and communication. In the United States, the American Academy of Pediatrics and the American College of Critical Care Physicians support family and patient-centered care and recommend open visitation for both adult and pediatric patients [2]. These recommendations are based on data demonstrating both improved satisfaction and decreased stress of both patients and their family members when open visitation policies are adopted [3–8], as well as data demonstrating no adverse health effects of open visitation with some suggestion of improved outcomes when open visitation is facilitated [9–13].

Family presence also improves patient physiology as demonstrated by multiple empirical studies. Presence of family members improves physiologic parameters in the maternity setting, including epinephrine levels, uterine blood flow, and fetal oxygenation [14]. Family presence also has positive physiologic effects on ICU patients and has been shown to lower intracranial pressure, improve heart rates, and improve blood pressure [15]. Multiple studies in cardiac intensive care units have also demonstrated improved hemodynamic changes associated with family visits suggesting a calming effect on patients [14, 16, 17]. 

Unfortunately, there are multiple barriers to families visiting their loved ones who are hospitalized. Potential barriers include time constraints due to the necessity of continuing to work to maintain income or the need to care for other children or family members at home. Further, geographical distance may be a significant barrier for many families. Specialty care is typically regionalized for increased efficiency and quality [18–20], resulting in many patients being transported to tertiary or quaternary medical centers that may be quite distant from their homes. This is particularly true in the case of pediatric patients due to the relative paucity of pediatric referral centers and the even greater need to regionalize care [1, 21]. 

To address patient and family anxiety, stress, and well-being, we developed a videoconferencing program (Family-Link) to link patients with their families and friends when they are unable to visit the hospital on a regular basis. A chart audit was conducted to determine the nature of sessions organized by the Family-Link program.

## 2. Methods

### 2.1. Service Requirements

“Family-Link” connections rely on dual video conferencing units that communicate over simple telephone lines or internet so that patients can simultaneously see and hear other family members and friends. Specifically, the first phase of Family-Link worked through connecting a normal television set with a ViaTV videophone (Vizufon—C&S Technology, Inc., Korea; http://www.cnstec.com/eng/company/01_company_info.html/), which transmitted images on a television screen, while audio is shared through the videophone. The second and current phase of Family-Link program uses laptop computers with built-in or plug-in webcams. One videophone or computer is placed in the patient's room while a second one goes home with the child's loved ones so that they can communicate and see one another. When using the computers and the internet, we use a third-party server to create a HIPPA compliant secure connection (Click To Meet, Radvision, Ltd, Tel Aviv, Israel; http://www.radvision.com/Corporate/AboutUs/). 

Our telemedicine technical team tracks where the devices are and are available 24/7 over telephone to the family (both at the bedside and home) in case technical support is required. Hardware maintenance and project management are provided free of charge by the previously existing University of California Davis Medical Center and the Center for Health and Technology. The goal of Family-Link is to provide a low-cost, technologically simple form of communication to support the patient during hospitalization.

This study and chart review was approved by the Human Subjects Review Committee at the UC Davis Medical Center.

## 3. Results

Between 2006 and 2010, the Family-Link service was provided to 36 families. In the majority of cases, the Family-Link was used to connect a patient in their hospital room to their family's home; however we also used the Family-Link to connect patients to friends at community functions, to schoolmates in the classroom, and even to other family members who were hospitalized at other facilities.

Table 1 shows all of the Family-Link cases that occurred from 2006 through 2010. The median age of patients involved in the program was 10 years (Inter Quartile Range, IQR: 4–14 years). Although the Family-Link service was originally introduced for pediatric patients, we found it very useful for one male patient in his late thirties (see detailed case description below). The most common reasons for using the telehealth service were related to the logistical challenges of travelling to and from the hospital—principally due to distance, time, family commitments, and/or personal cost. The median distance between the hospital and families involved in the program was 88 miles (IQR: 43–201 miles).

### 3.1. Selected Case Studies

#### 3.1.1. Case  1

TG was in his late 30's and lives approximately 1,400 miles from the UC Davis Medical Center. He taught high school science and was a vital member of his community with many friends. In June, 2006, TG was driving his car when a deer ran across the road. TG was driving about 75 miles per hour, and he was unable to avoid hitting the animal. The accident did not seem to cause any significant injury to TG, and he was feeling fine after a day or two. In July, 2006, he was playing Frisbee with his son at a recreation area that had a shallow pond. TG was standing in 6 inches of water when he abruptly lost consciousness, collapsed, and nearly drowned. His wife, a nurse, found him apneic and pulseless. She performed CPR, and 911 was called. When TG reached his local hospital, he was stabilized and they performed an anterior cervical corpectomy of C5-C6 and posterior arthrodesis of C4–C7. After a week of acute stabilization treatment, TG was transferred to the University of California Davis Medical Center in Sacramento, California for intense acute rehabilitation therapy.

TG's friends wanted to show him how much they cared, so they decided to hold a fundraiser to help him pay his medical expenses. Over 2,000 people came to show their support in his hometown. The Family-Link program was contacted and made it possible for TG and his friends to talk to and see one another. We set up a Family-Link terminal in a portable tent at the fundraiser, and while sitting in his hospital bed in Sacramento, TG was able to visit with friends and family in Glasgow for three hours. After the event, TG was overjoyed and stated that being able to see and talk with his friends and family raised his spirits tremendously and gave him even more motivation in his rehabilitative therapies.

#### 3.1.2. Case  2

CR was a 15-year-old girl living in a town approximately 200 miles north of the UC Davis Medical Center. CR was a high school sophomore living with her mother, brother, and great aunt. CR was spending time with her friends one autumn day after school when a driver lost control of his vehicle and careened onto the sidewalk. CR was pinned between the car's bumper and the brick wall behind her. She was taken to a local hospital and then immediately transferred to UC Davis for acute stabilization. The injuries were localized to CR's legs, however she required an above-knee amputation on the left, and her right leg required several procedures including repair of the artery and knee.

Her friends wanted to visit CR, and she too wished to see them, but 200 miles is a long way to travel, particularly when you are a teenager. The town she is from is small, with a population of approximately 3,500 people, so everyone in town is well connected. CR was particularly close to her classmates, about 30 other kids who had spent their lives growing up together. Our Family-Link coordinator contacted the school, and a special assembly was scheduled. During the lunchtime assembly, CR was connected to her classmates with real-time videoconferencing. CR told us that being able to see and talk to her friends made her very happy. Indeed, the hospital staff noted that CR's mood was substantially improved after this experience. CR continued to use Family-Link over the next four weeks of her recovery to interact with her high school friends. She expressed that the Family-Link made her hospital stay much more bearable.

#### 3.1.3. Case  3

MU was a 15-year-old girl when she suffered a severe spinal cord injury. MU lived with her parents and her older brother in a town 80 miles from the UC Davis Medical Center. One evening, while she and her brother were driving to a function, they were involved in a high-speed collision, and MU was ejected from the vehicle. A local hospital diagnosed spinal cord compression with lower extremity paraplegia, and MU was emergently transferred to UC Davis for decompressive laminectomy and spinal stabilization. MU's surgery was a success, and she quickly improved.

Unfortunately, MU's brother's injuries were fatal, and he was declared dead on the scene. While MU was being cared for at our hospital, her parents were arranging her brother's funeral. MU was very close to her brother, her only sibling. MU asked if she could leave the hospital to attend the funeral, but the neurosurgeons felt that she was not stable enough to make the trip home.

The Family-Link coordinator worked with MU's parents. She contacted the family's church and explained the Family-Link project. The church agreed, and we provided a link allowing MU to attend her brother's funeral from her hospital bed. While MU's parents attended the funeral in person, MU was not alone. A very close family friend and our Child Life Specialist stayed at her side. MU was supported, and although distraught by the loss of her brother, she felt some measure of peace being able to attend his funeral even if only via Family-Link. Today, MU has recovered remarkably well. She now walks without difficulty and complains of only mild back pain. Being able to participate in her brother's funeral remains one of her strongest memories of her hospitalization.

## 4. Discussion

Hospitalization is a very stressful time for the patient and their family. This stress is increased when the admission is to an ICU and is heightened when the family and friends are not able to be present to support their critically ill or injured loved one. Family-Link is a simple videoconferencing program that provides a solution to help address these barriers and provides the opportunity for remote families and friends to visit their critically ill or injured loved one. The simple and relatively inexpensive communication technology (phone lines and/or internet) may allow for better family coping which follows the Institute of Medicine's recommendations of a patient-centered model of care in which the “health care delivery systems provide for the physical comfort and emotional support of patients and family members.” [17].

### 4.1. Value of Family Presence

The ability of the hospitalized patient to interact with previously established support providers (e.g., spouse, children, parents, siblings, extended family, friends, or clergy) assists in adapting to the unfamiliar hospital environment thereby improving the patient's psychological state. Family presence has positive effects on a patient's perception of pain, loneliness, and fear [22]. Further, because having family members and friends present at the bedside provides an opportunity for hospital staff to witness first-hand the patient's support system, family presence improves delivery of care [23].

When a child is admitted to the ICU, the stress on the child and their parents is tremendous [24]. Parents often feel particularly distressed by the lack of control they have over their child's care [12]. Many parents note that at home they are primarily responsible for taking care of their children, keeping them safe, and nursing them to health during illness. In the ICU, parents are often stripped of their ability to provide direct care and are uninvolved with many of the day-to-day decisions. Parents often describe a feeling of helplessness [13], and this alteration in parental role is the major cause of parental stress during their child's ICU admission. Notably, the ability to see their child frequently is essential for most parents of children in the ICU [25].

### 4.2. Family Presence in the NICU

The use of communication technology to assist in family presence has been previously used in the Neonatal Intensive Care Unit (NICU). Several programs in the United States incorporate both videoconferencing and the World Wide Web technology for improving communication in the NICU among family members and members of the health care team (Angel Eye, University of Arkansas, Little Rock, AK; Baby CareLink, Beth Israel Hospital, Boston, MA; Neonatal Examination and Management Online, NEMO, University of Queensland, Brisbane, Australia). During an infant's hospitalization, family members are able to see the newborn, along with a web site that included a daily clinical report, a message center, a pictorial journal, and/or a clinical information section. These systems also allow for virtual house calls and remote monitoring after discharge. One NICU program was evaluated with families of very low birth weight infants during and after their hospitalization, and the investigators found significantly improved family satisfaction. The authors reported that this technology further supported the educational and emotional needs of families and may have resulted in earlier discharge to home with improvements in the coordination and efficiency of care in the postdischarge period [23]. The Family-Link program allows for two-way video communication so that there are direct benefits of communication for both the family as well as the patient.

Another NICU employs a one-way, real-time video feed from the neonate's bed to a password-protected website accessible only to family and selected hospital staff. All mothers who participated in the program expressed that viewing their infant twice daily relieved anxiety and aided in connecting with their baby and thus enhanced bonding despite the distance barriers. The technology also provided family members from other states to see the baby, further connecting the family [26]. Home videoconferencing has also been shown to be successful in the postoperative management of children recently discharged with complex congenital heart disease [15, 27] as well as other complex diagnoses [15, 28–30] with a high degree of parent satisfaction [31].

### 4.3. Technology and Replication

Videoconferencing between a hospitalized patient and remote family and/or friends can be accomplished in a multitude of ways. The basics require some video-audio device at the patient and family locations and some telecommunications technology connection to connect the video-audio devices. Family-Link employs simple videoconferencing units that use standard telephone lines or internet for telecommunications. For telephone lines, there are several commercially available models that offer built-in video and real-time communication at 30 frames per second, an industry standard that provides easy to watch video. 

Videoconferencing between a hospitalized patient and remote family and/or friends can also be accomplished using broadband internet and standard webcam-based technologies. Such models can use simple webcams or more expensive videoconferencing units, computer video monitors, and the internet as a means of telecommunication. While high speed internet can provide good quality audio-visual connections, the internet provides variable connection speeds and can result in inconsistent telecommunications and connection quality. Access to internet may also not be universally available to the remote family/friends, have additional security issues, may be obstructed by hospital firewalls, and can be more complicated and expensive to set up and to maintain. In addition, whether using simple telephone lines or internet, personnel are required to identify patients in need, set-up the equipment, trouble shoot connectivity problems, and maintain an inventory of equipment.

Any such system carries certain ethical and legal responsibilities. With the recent implementation of Health Insurance Portability and Accountability Act (HIPAA) regulations in the United States, providers must be cautious when providing information to individuals via Family-Link just as they would when providing information in person. Patients and/or parents may wish to limit information or have information given only to specific individuals. Further, in the case of pediatric patients, care must be taken to ensure that direct communication with the patient is appropriate. When Family-Link connections are open continuously, patients may be viewed without their immediate knowledge, therefore specific directions should be sought to determine when the connection is active and who may “turn on” the system. Further, as with in-person visits to the ICU or ward, children (whether a child of the patient, siblings of pediatric patients, or other minor “visitors”) should be prepared for what they will see when visiting their critically ill relative. Child life specialists, social workers, and nurses may need to help parents prepare children or even “meet” with children, either in person or via Family-Link, to help prepare them in advance to minimize emotional trauma.

## 5. Conclusions

The essential role that families and friends play in the care and recovery of hospitalized patients is becoming increasingly recognized. The nature of the hospital and ICU environments as well as the time and barriers to visiting the hospital often limits family-friend participation. Videoconferencing provides a practical solution to some barriers that may limit family presence and participation. Future studies of programs such as the Family-Link project should examine the cost-effectiveness of this approach and patient care outcomes. Further, as more hospital wards and ICUs include family members on daily rounds, as recommended by the American Academy of Pediatrics and the American College of Critical Care [2], Family-Link type projects may facilitate this practice when parents or family members cannot be physically present in the hospital. The ultimate goal is to use communication technologies to improve patient centered care.

## Figures and Tables

**Table 1:** Description of Family-Link cases (in order of use).

Patient age	Reason for admission	Need-unique use for family-Link	Length of stay (days)	Distance (miles)
12 years	Multidrug resistant pulmonary tuberculosis	Single father unable to visit because of limited transportation and finances. There were also 8 other siblings at home. Father and older brother provided primary incomes and were unable to take time away from work	45	50

15 months	Leukemia	Parents and siblings living far from Sacramento	13	50

17 years	Quadriplegia admitted for rehabilitation	Parents and siblings living far from Sacramento	30	220

17 years	Chemotherapy	Divorced parents each had a unit to communicate with daughter because of distance	79	94

4 years	Pneumonia	Single mother not able to spend time with patient and distance	12	50

12 years	Severe traumatic brain injury	Large extended family. Family-Link also utilized to allow family members to participate in patient's rehabilitation regimen to prepare for home therapy	27	30

14 years	Myasthenia gravis	Set up for ill grandfather and other family members who were unable to visit on a regular basis and wanted to be able to check on patient's progress	208	10

38 years	Spinal cord injury	See Section 3.1.1	68	1,400

14 years	Tuberculosis, isolation	Family-Link utilized to allow communication for two teenage friends both in isolation and in adjacent rooms	23	76

14 years	>90% total body burn injuries	Patient able to attend 8th grade graduation using Family-Link	195	750

15 years	Struck by motor vehicle, multiple trauma.	See Section 3.1.2	21	210

11 years	Leukemia and dilated cardiomyopathy	Extended family wanting to visit but difficult because of long distance	12	150

4 years	Spinal cord injury	Recently separated parents were unable to visit at the same time because of domestic issues and conflicting work schedules	46	217 (father) 94 (mother)

16 years	Pneumonia and respiratory isolation	The patient and family lived in Nevada. Mother was only source of income and unable to take time off from work	59	127

18 months	Polycystic kidney disease status postrenal transplant	Family-Link was set-up for patient to view his father and sibling at home who were unable to spend time with him due to distance	95	144

3 years	Motor vehicle accident with anoxic brain injury	The patient's mother wanted to withdraw medical support, but was concerned because the patient's father was incarcerated and unable to be involved in the decision-making process. Using Family-Link, a connection was established with the patient's father in prison. He was able to see his son's unresponsiveness and was able to provide further support to help with the decision to limit medical care and withdraw support	22	65

15 years	Spinal cord injury with lower-extremity paraplegia	See Section 3.1.3	32	65

6 years	Pneumonia	Dad, siblings, and grandmother not able to visit often because of limited transportation options	9	25

10 years	Acute disseminated encephalomyelitis	Family from city located 5 1/2 hours. One parent stayed at home with other children and visited daily	19	323

6 months	Leukemia	Young sibling at home, grandparents in Los Angeles	14	387

10 years	Rehabilitation	Extended hospital stay and parent must stay with siblings at home	28	24

2 years	Cystic fibrosis	Siblings staying with grandparents, unable to visit	21	166

2 years	GI surgery	Sibling and father at home, unable to visit during the week	23	73

3 years	Pharyngeal cellulitis, dehydration	Siblings and father at home. Father had to work and could not bring other siblings to visit	5	36

3 years	Leukemia	Family (aunt and cousins) located in Philadelphia, unable to visit	6	2,458

10 years	Traumatic brain injury	Parent worked full time and unable to visit hospital frequently	10	30

7 years	Sepsis	Sibling at home with grandparent, unable to visit	12	17

8 years	Removal of brain tumor	Family support network located in Jordan, unable to visit	11	7,448

10 years	Pneumonia	Siblings unable to visit due to distance	7	170

12 years	Midgut volvulus	Father unable to visit due to distance and work schedule	5	174

9 years	Trauma, impaled buttocks	Siblings staying with friends, unable to visit	7	40

10 years	Femoral fracture	Family at home, distance too far to travel	6	82

10 years	Perforated appendicitis	Ill family member (aunt), unable to visit hospital	13	25

15 years	Ladd's procedure	Grandmother unable to visit	8	1,458

14 years	Hematochezia, inflammatory bowel disease	Siblings and parents unable to visit due to distance	5	136

13 years	Multisystem trauma	Grandparents and extended family located in Pennsylvania	25	1,485
